# Academic procrastination; it may be more than meets the eye

**DOI:** 10.30476/jamp.2020.86256.1231

**Published:** 2021-01

**Authors:** LAILA DANESH, HOSNA SHERZAI, AMINA MUSHTAQ, MARIAM AL-JUMAILY, SANA HASHEMI, DOST JABARKHYL

**Affiliations:** 1 King's College London School of Medicine, Strand, London, WC2R 2LS, United Kingdom

**Dear editor**

We read, with great interest, the article by Hayat et al ( 1 ). We are glad that such a pertinent issue is finally being explored. As medical students, we are aware of how vital productivity is; therefore, this paper is key to helping us find reasons and answers to any hindrances in our productivity. Moreover, it was interesting and shocking to learn that procrastination behaviours are not only linked to lower academic achievements, but also higher course withdrawal. Having said that, we would like to point out some discrepancies in the research methods and results. 

The authors acknowledged that because the study was carried out on medical students, the results are consequently less generalizable to all students, thus decreasing its external validity ( 2 ). However, we would like to argue that the results are also not generalizable to, or representative of, medical students either. This is because the sampling method selected -convenience sampling- may present higher levels of inapplicability and invalidity due to the method being subject to selection bias. This is because the sample method has two main inherent flaws:

1) It magnifies the unconscious biases of the researchers as only certain cohorts are convenient for them to sample. Moreover, despite the somewhat balanced gender ratio, there are other confounding variables that may have affected the results which we shall illustrate below.

2) Certain personality traits, eg extraversion, are also amplified in a non-random sampling method ( 3 ). This can explain the low response rate of 71% as those with more introverted tendencies may not have completed it. 

Moreover, Type A personality traits [e.g. being goal driven, organized and a ‘workaholic’ ( 4 ) ] are overrepresented in medical schools ( 5 ) compared to the general population which contradicts the finding that there is a high level of procrastination amongst students. One can assume logically that those with Type A personalities are less likely to procrastinate as it is seen as ‘time wasting’. It is possible that both are true and medical schools have a high percentage of procrastinators and Type A personalities. However, to confirm this we suggest that more in-depth research is needed to see the prevalence of personality traits and how they affect the behaviour of students in regard to Internet use and productivity. 

Whilst this study accounted for and differentiated between students living at home and those living in college dormitories, there are other confounding factors that the authors failed to account for and explore. For example, medical students are known to have higher rates of depression and anxiety ( 6 ) compared to the general population. Known symptoms of depression include anhedonia and anergia ( 7 ), which can present as procrastination and that affects one’s ability to be productive; we often see colleagues struggling with mental health issues mask it under the guise of procrastination on the Internet. It would be interesting to see if there was a correlation between students diagnosed with the mentioned condition and their level of procrastination and Internet addiction. Exploration of these variables may help us find a causative link between the correlations.

We agree with the authors that a longitudinal study is needed. However, this must include an emphasis on recruiting a representative sample using a verified measures of personality types, productivity and considering the aforementioned confounding factors to increase the internal and external validity. 

As medical students, we are just as eager to find out what the cause and solution to this issue is, so we welcome all further research into it. We acknowledge that whilst the Internet might be the tool of choice for procrastinators, it is also vital for our education experience. 
